# Evaluation of Mean Percentage of Full-Length SMN Transcripts as a Molecular Biomarker of Spinal Muscular Atrophy

**DOI:** 10.3390/genes13101911

**Published:** 2022-10-20

**Authors:** Marianna Maretina, Anna Egorova, Kristina Lanko, Vladislav Baranov, Anton Kiselev

**Affiliations:** 1 Department of Genomic Medicine, D.O. Ott Research Institute of Obstetrics, Gynecology and Reproductology, Mendeleevskaya Line 3, 199034 Saint-Petersburg, Russia; 2 Department of Clinical Genetics, Erasmus MC University Medical Center, Dr. Molewaterplein Street 40, 3015 GD Rotterdam, The Netherlands

**Keywords:** spinal muscular atrophy, SMN transcripts, molecular biomarker, antisense oligonucleotides, SMN expression, *SMN2* gene

## Abstract

The elevation of SMN transcript and protein level remains the principal aim of SMA therapy. Still, there is no standard molecular biomarker for the assessment of its efficacy. In the current study, we tested three methods of SMN transcript level measurement using real-time RT-PCR, quantitative fluorescent RT-PCR, and a semiquantitative RT-PCR gel densitometric assay. We examined several potential mRNA-based biomarkers and examined their sensitivity and reliability by comparing the obtained values in peripheral blood mononuclear cells of SMA patients, SMA carriers, and healthy individuals. We found that the mean percentage of full-length (FL-SMN) transcripts relative to the total sum of FL-SMN and exon 7-deleted (Δ7 SMN) transcripts detected by semiquantitative and quantitative fluorescence RT-PCR differed significantly between the three analyzed groups. The relevance of this biomarker was proven in an SMN2-targeting therapeutic experiment. We showed that the values of the biomarker changed significantly in SMA fibroblast cell cultures after treatment with therapeutic antisense oligonucleotides targeting the ISS-N1 site in intron 7 of the *SMN2* gene. The obtained results indicate the convenience of using the mean percentage of FL-SMN transcripts determined by semiquantitative and quantitative fluorescence RT-PCR as a putative biomarker for the assessment of SMA therapy efficacy in vitro.

## 1. Introduction

Spinal muscular atrophy (SMA) is one of the most severe hereditary neuromuscular diseases, with an incidence of about 1 per 6000–10,000. SMA is an autosomal recessive disorder that results from mutations in the survival motor neuron gene *SMN1*, located on chromosome 5q13 [1]. This gene has a highly homological copy—the *SMN2* gene that differs from *SMN1* by several nucleotides. Only the substitution of C to T in exon 7 of the *SMN2* gene is functionally significant and disrupts pre-mRNA splicing, resulting in a lack of exon 7 in most SMN transcripts [2]. The protein translated from the aberrantly spliced mRNA is truncated and unstable. In SMA patients, the *SMN2* gene is the only source of SMN protein, and the number of its copies correlates with disease severity, which makes *SMN2* the main modifier of spinal muscular atrophy [3]. Numerous additional molecular factors can affect SMA pathways, thus increasing phenotype heterogeneity [4].

Currently, the correction of *SMN2* gene splicing is one of the most effective approaches to SMA therapy, along with the delivery of a functional copy of the *SMN1* gene into the cells of SMA patients [5]. The efficacy of existing drugs to treat SMA is rather individual and may depend on the *SMN2* gene copy number or other factors [6]. Therefore, there is an unmet medical need to have a reliable biomarker that enables the assessment of the efficiency of therapy in a particular patient with SMA. In addition, the existing drugs for SMA therapy demonstrate a number of side effects [7,8]. Moreover, studies demonstrated that a combinatorial approach to SMA therapy could increase the efficiency of the treatment. To develop alternative approaches to treat the disease, accurate methods are needed to assess their clinical efficacy in cellular models.

Different tests for measuring motor function in SMA patients are able to determine the drug efficiency only after a long time period. Frequently, these indicators are not suitable for preclinical trials, e.g., when testing the potential drugs in vitro. Thus, it is necessary to have additional molecular biomarkers that make it possible to assess the effect of the drug quickly and accurately in a cellular model of the disease or in heterogeneous samples of SMA patients.

To date, there is no universal molecular biomarker of SMA and no optimal method to accurately estimate its value. Among potential biomarkers studied to date are the relative level, percentage, and ratio of full-length (FL) and truncated (Δ7) SMN transcripts; SMN protein level; and the number of SMN-associated nuclear bodies–gems [9,10,11,12,13,14,15]. Before testing any biomarker, it is necessary to check its sensitivity and reliability. Such analysis can be performed on samples of SMA patients, SMA carriers, and healthy individuals. Since these three groups of individuals are characterized by different *SMN1* and *SMN2* gene copy numbers, the identification of statistically significant differences in the values of FL and Δ7 SMN transcripts between these groups may indicate the reliability of the selected biomarker and the method for its assessment.

Here, we developed three approaches to measure the SMN transcript level based on real-time RT-PCR, quantitative fluorescent RT-PCR, and semiquantitative RT-PCR gel densitometric assay and validated the best biomarker found in an SMN2-targeting therapeutic experiment in cells derived from SMA patient.

## 2. Materials and Methods

### 2.1. Materials

This study was performed using large-scale research facility #3076082, “Human Reproductive Health”, at the D.O. Ott Research Institute of Obstetrics, Gynecology and Reproductology (Mendeleevskaya Line 3, 199034 Saint-Petersburg, Russia). Venous blood samples were collected from SMA patients (2 type I, 22 type II, 7 type III, and 1 type IV), 44 carriers of the disease, and 31 healthy individuals. For PBMCs sedimentation, 2 mL of whole blood was mixed with 12 mL of 0.74% NH_4_Cl lysis buffer, incubated for 30 min at +4 °C, and then centrifuged for 20 min at 1500 rpm. After the removal of supernatant, PBMCs were resuspended in RNA-stabilizing solution IntactRNA (Evrogen, Moscow, Russia) and frozen at −70 °C.

Primary fibroblast cell culture was obtained from skin biopsy of patient with SMA type II. Cells were maintained at 37 °C in 5% CO_2_ in DMEM containing L-glutamine and 4.5 g/L glucose (Biolot), supplemented with 10% FBS (Gibco) and penicillin–streptomycin (Biolot) (penicillin 100 U/mL, streptomycin 100 μg/mL), as described previously [16].

Informed consent was obtained from all the participants of the study. All the participants were analyzed for presence of deletion in *SMN1* gene, and the number of *SMN2* gene copies was determined as described previously [3]. Age in the studied groups ranged from 1 to 33 years in SMA cohort; from 26 to 47 years in the carrier cohort; and from 20 to 48 in non-carrier cohort. Distribution of *SMN2* gene copy numbers between studied cohorts is shown in Figure S1.

### 2.2. RNA Isolation and cDNA Synthesis

To wash out the IntactRNA solution, PBMCs were mixed with PBS in a 1:3 ratio and centrifuged for 10 min at 10,000 rpm. After the removal of supernatant, the pellet was washed again with 100 µL of PBS and centrifuged for 3 min at 10,000 rpm. The liquid was removed; the pellet was resuspended in 250 µL of TRIzol reagent and incubated for 5 min at RT. Then, 100 µL of chloroform was added, and the contents of the tube were mixed, incubated for 3 min, and centrifuged for 15 min at 14,000 rpm. The upper phase was transferred to an empty tube, and RNA was precipitated with 125 µL of isopropanol. The tube was stored at −70 °C overnight. The next day, tubes were centrifuged for 20 min at 10,000 rpm (+4 °C), and the supernatant was discarded. The precipitate was washed with 125 μL of 70% ethanol and centrifuged for 3 min at 14,000 rpm. The supernatant was discarded, and RNA pellet was left to dry for 1 h. To dissolve the RNA, 40 μL of DEPC-treated H_2_O was added to the pellet and incubated for 40 min at room temperature. To extract RNA from fibroblasts cultured in 24-well plates, cells were washed twice with 200 µL of PBS and removed from the culture surface by incubation in 200 µL of Trypsin–Versene Mixture (1:3) for 10 min at 37 °C. Then, 300 µL of PBS was added to wells, mixed, transferred to tubes, and centrifuged for 10 min at 2200 rpm. The supernatant was discarded, and 125 µL of TRIzol reagent was added to the residue. RNA isolation was performed according to the method described above in half volume.

About 1 μg of total RNA was reverse-transcribed using first strand cDNA synthesis kit with random primers (Sileks, Moscow, Russia) according to the manufacturer’s protocol.

### 2.3. Real-Time PCR

Real-time PCR was carried out using Eva Green PCR kit (Syntol, Moscow, Russia) in a Rotor-Gene 3000 thermal cycler (Corbett Life Science, Mortlake, Australia). A total of 2.5 µL of cDNA was amplified in a total volume of 25 µL, which included 2.5 μL 10× Eva Green buffer, 4 µL MgCl_2_ (25 mM), 2.5 µL dNTPs (2.5 mM), 1 µL of each primer (5 μM), 0.3 μL SynTaq polymerase (5 U/μL), and 11.2 μL ddH_2_O. Full-length and Δ7 SMN transcripts, as well as *H3B* and *GAPDH* reference gene transcripts, were amplified with primers described previously [3]. The reaction conditions were 95 °C for 3 min, 45 cycles of 95 °C for 20 s, 57 °C for 30 s, and 72 °C for 30 s. Each cDNA sample was amplified in duplicate.

### 2.4. Semiquantitative and Quantitative Fluorescence RT-PCR

A total of 1 µL of cDNA was added to the PCR mix, which included 1 µL of 10x PCR buffer with MgCl_2_, 1.25 mM dNTPs, 1 µM of each primer, and 5 U of Taq DNA polymerase (SibEnzyme, Novosibirsk, Russia). The following primers were used for full-length and Δ7 SMN transcripts amplification: SMN F 5′-GTCCAGATTCTCTTGATGAT-3′, complementary to SMN exon 6 region and SMN R 5′-CTATAACGCTTCACATTCCA-3′, complementary to SMN exon 8 region. For quantitative fluorescence PCR (QF-PCR), the forward primed was tagged with FAM. The amplification reaction was conducted at 94 °C for 4 min, n cycles of 94 °C for 45 s, 50 °C for 45 s, 72 °C for 45 s, and final synthesis at 72 °C for 8 min. The number of cycles (n) did not exceed 26–28. Amplification of each cDNA sample was performed at least 2 times.

The amplification products obtained after semiquantitative RT-PCR were separated on 6% polyacrylamide gel, then stained in ethidium bromide solution (0.5 μg/mL) and photographed on a transilluminator in transmitted ultraviolet light (wavelength 380 nm). The luminescence intensity of the amplification products was assessed using ImageJ (NIH, Bethesda, MD, USA).

To visualize the results of QF RT-PCR, 1 μL of the PCR product was mixed with 12 μL of formamide (MCLAB, CA, USA) and 0.25 μL of the molecular-weight marker LIZ500 (Applied Biosystems, CA, USA), and then the fragments were separated using an ABI 3130xl capillary electrophoresis instrument at 60 °C. The analysis of the results was carried out using GeneMapper software (Applied Biosystems, CA, USA).

### 2.5. Transfection

Primary SMA fibroblasts were obtained previously from SMA type II patient [16] and plated 24 h prior to transfection on 24-well plate in DMEM containing L-glutamine and 10% FBS so that on the day of transfection, the cells reached ~50% confluency. Cells were transfected with RNA oligonucleotides using XtremeGENE transfection reagent (Roche, Paris, France) according to manufacturer’s recommendations. 3UP8 antisense oligonucleotide restoring *SMN2* exon 7 splicing and F8 oligonucleotide that served as a negative control previously reported by Singh and colleagues was used in the experiment [9]. Then, 10:1 and 5:1 ratios of XtremeGENE (μL) to RNA (μg) were tested. Each RNA/carrier complex was added in duplicate. Transfected fibroblasts were incubated for 4 h at 37 °C in 5% CO_2_; then, the medium was changed to that containing L-glutamine, FBS, and an antibiotic, and cells were incubated for 48 h in CO_2_ incubator. After, fibroblasts were removed from the culture surface, and RNA was isolated as described in the corresponding section. All transfections were repeated at least 2 times.

## 3. Results

### 3.1. Determination of Relative Full-Length and Δ7 SMN Transcripts Level in Patients, SMA Carriers, and Healthy Individuals by Means of Quantitative Real-Time PCR

**Figure 1:**
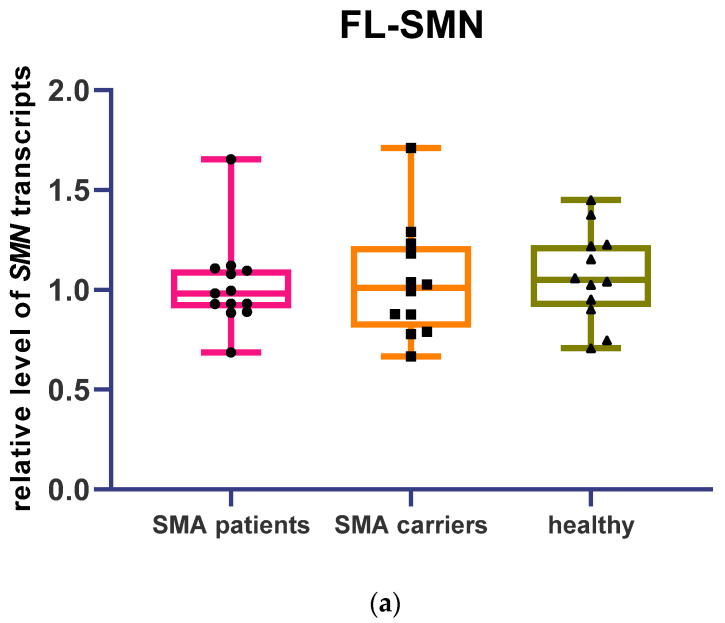

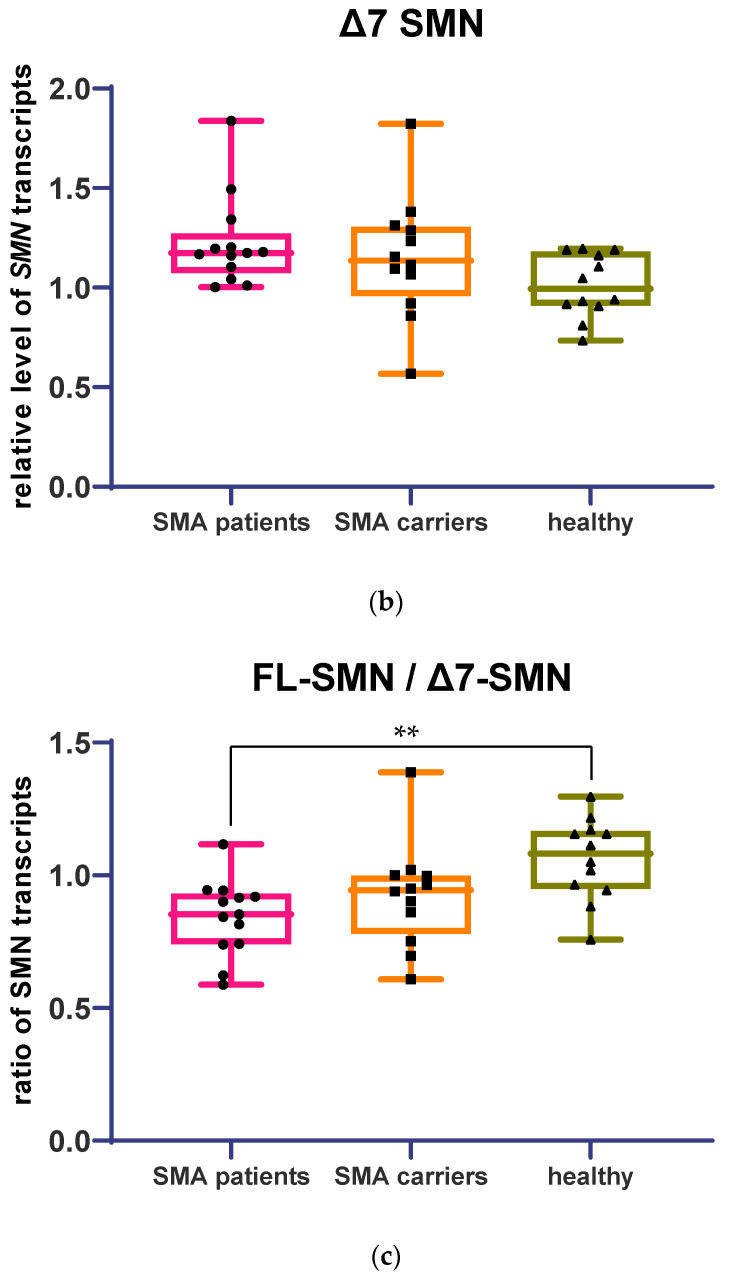
Level of full-length and Δ7 SMN transcripts relative to *H3B* and *GAPDH* transcripts obtained by real-time PCR in SMA patients’ (**a**), SMA carriers’ (**b**), and healthy individuals’ blood cells (**c**). Ratio of FL to Δ7 SMN transcripts (significantly higher in healthy individuals’ cells compared to SMA patients’ cells, **-*p* < 0.01 determined by Kruskal–Wallis test with Dunn’s follow-up test).

Levels of full-length and Δ7 SMN transcripts were measured by real-time PCR in cDNA samples of 13 patients, 12 SMA carriers, and 12 healthy individuals. Relative levels of FL and Δ7 SMN transcripts were determined based on cycle threshold (Ct) values obtained after the real-time run. First, the corrected Ct value was calculated by the formula Ct_cor_ = E^(Ctc − Ct)^, where E is the efficiency of the reaction, Ctc is the mean Ct value of calibration samples, and Ct is the cycle threshold of fluorescence level for the analyzed sample. Next, the normalization factor (F_norm_) was calculated as the geometric mean of the corrected Ct values obtained from the *H3B* and *GAPDH* reference genes. Finally, the normalized Ct value was calculated by the formula Ct_norm_ = Ct_cor_/F_norm_. No statistical difference was observed in the relative level of FL and Δ7 SMN transcripts between patients, SMA carriers, and healthy individuals (Figure 1a,b). At the same time, a significant difference in the ratio of FL to Δ7 SMN transcripts was detected between SMA patients and healthy individuals (Figure 1c) (median FL/Δ7 ratios of SMN transcripts were 1.08 for healthy individuals, 0.95 for SMA carriers, and 0.85 for SMA patients).

### 3.2. Full-Length and Δ7 SMN Transcripts Percentage Detected in Patients, SMA Carriers, and Healthy Individuals by Semiquantitative and Quantitative Fluorescence RT-PCR

For RT-PCR, we designed primers complementary to exon 6 and exon 8 of *SMN* cDNA that enabled the detection of FL and Δ7 SMN transcripts simultaneously. Because smaller fragments are known to have higher a efficiency of amplification, the number of PCR cycles was chosen to analyze the PCR products within the exponential phase.

**Figure 2:**
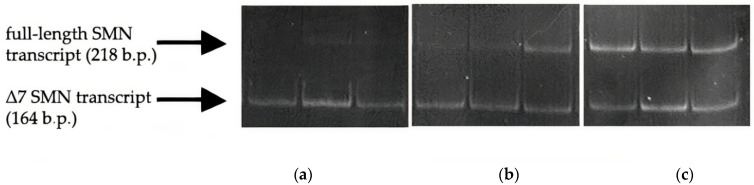
PCR products of full-length (218 b.p.) and exon 7-deleted (164 b.p.) SMN transcripts from SMA patients’ (**a**), SMA carriers’ (**b**), and healthy individuals’ (**c**) blood cells on PAA gel electrophoresis.

An example of the raw gel electrophoresis data of PCR products of samples of healthy individuals, carriers of the disease, and patients with SMA is given in Figure 2.

**Figure 3:**
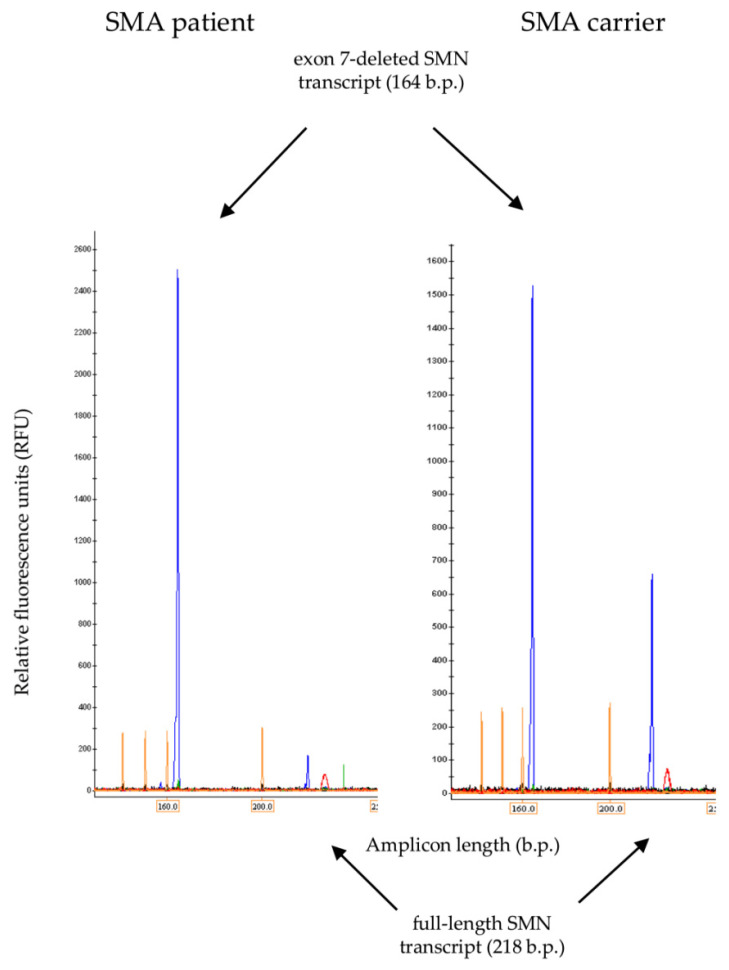
Typical appearance of fluorescence peaks corresponding to full-length and Δ7 SMN transcripts obtained in QF-PCR analysis for SMA patient and SMA carrier samples.

The FL and Δ7 SMN transcripts percentage was calculated based on the fluorescence intensity of the bands corresponding to the amplicons of these transcripts relative to the background in ImageJ software. The percentage values obtained for FL and Δ7 SMN transcripts were verified by means of QF-PCR. An example of fluorescence peaks obtained in QF-PCR analysis for SMA carrier and SMA patient samples is given in Figure 3. The results obtained by the two methods highly overlapped (correlation coefficient of 0.95) (Figure S2).

**Figure 4:**
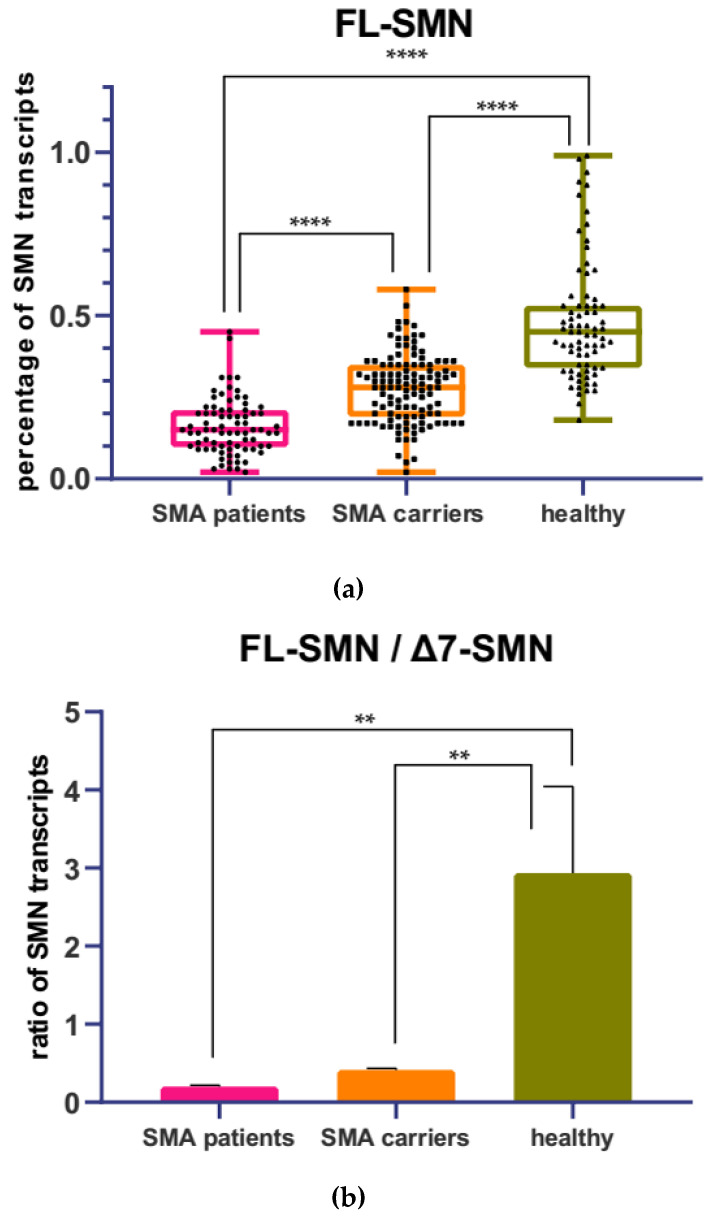
Mean percentage of FL-SMN transcripts (**a**) relative to the total sum of FL-SMN + Δ7 SMN transcripts in SMA patients’, SMA carriers’, and healthy individuals’ blood cells obtained by means of semiquantitative RT-PCR. ****-*p* < 0.0001 determined by ANOVA. Ratio of FL-SMN to Δ7 SMN transcripts (**b**). **-*p* < 0.01 determined by ANOVA.

The level of *SMN* transcripts was determined by means of semiquantitative RT-PCR in 107 people, including 32 SMA patients, 44 SMA carriers, and 31 healthy individuals. For each sample, the mean value of the FL-SMN percentage was obtained, which was calculated as the ratio of the values obtained in the ImageJ program for full-length transcripts to the total sum of the values of the FL-SMN + Δ7 SMN transcripts. The mean percentage of FL-SMN transcripts was 0.16 for SMA patients, 0.28 for SMA carriers, and 0.48 for healthy individuals. ANOVA test with Tukey post-test revealed statistical differences between the three analyzed groups (Figure 4a).

At the same time, FL-SMN/Δ7 SMN transcripts ratio differed significantly between SMA patients and healthy individuals, as well as between SMA carriers and healthy individuals, but demonstrated no difference between SMA patients and SMA carriers (Figure 4b). The mean FL-SMN/Δ7 SMN ratio was 0.20 for SMA patients, 0.41 for SMA carriers, and 2.94 for healthy individuals.

### 3.3. Full-Length SMN Transcripts Percentage Increase after SMN2 Splicing Correction

**Figure 5:**
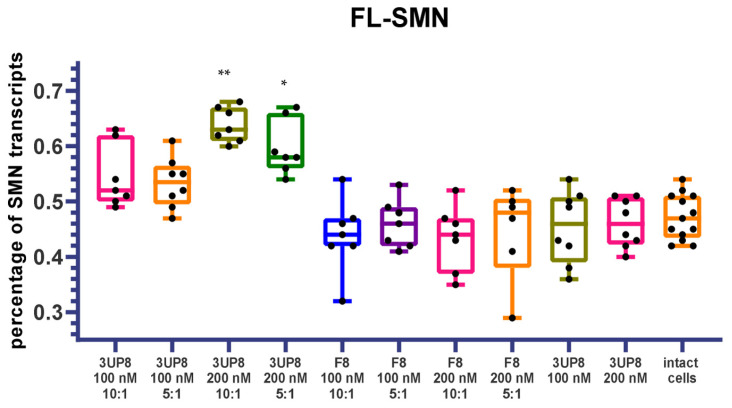
Mean percentage of full-length SMN transcripts in SMA fibroblast cell culture after delivery of therapeutic 3UP8 ASO and control F8 ASO. *-*p* < 0.05, **-*p* < 0.01 determined by Kruskal–Wallis test with Dunn’s follow-up test (comparison was performed relative to intact cells).

To evaluate the applicability of tested biomarkers for the assessment of therapy efficacy, we performed transfections of SMA fibroblast cell cultures with an antisense oligonucleotide (ASO) 3UP8 aimed at correction of *SMN2* splicing. A total of 100 nM and 200 nM concentrations of the therapeutic ASO were tested, as well as 10:1 and 5:1 ratios of X-tremeGENE:3UP8. Antisense oligonucleotide F8, previously demonstrated to have no therapeutic effect, was tested at the same concentrations as a negative control [9]. The efficiency of splicing correction was assessed based on full-length SMN transcripts’ percentage detected by semiquantitative RT-PCR and quantitative fluorescence RT-PCR because these methods demonstrated the most promising results. A dose-dependent increase in FL-SMN values was detected in cells treated with therapeutic 3UP8 ASO delivered with X-tremeGENE, while control F8 ASO or 3UP8 without transfection reagent did not induce splicing correction (Figure 5).

## 4. Discussion

In this study, we have developed and tested several methods for SMN transcript-level assessment. We applied these methods to examine the relative level, ratio, and percentage of FL and Δ7 SMN transcripts. Their sensitivity and suitability for usage as a biomarker of SMA therapy were determined based on the ability to detect differences between SMA patients, SMA carriers, and healthy individuals. Quantitative real-time PCR, semiquantitative, and quantitative fluorescence RT-PCR were among the tested methods. Results obtained after quantitative real-time PCR demonstrated statistical differences only between SMA patients and healthy individuals in the FL to Δ7 SMN transcripts ratio. Among the limitations of this method is the usage of different primer pairs for FL and Δ7 SMN transcripts detection, which can provoke variance in amplification efficacy and thus alter the real ratio of these transcripts. Moreover, the use of housekeeping genes as reference genes in expression analysis may compromise the accuracy of the results since it was shown that their expression varies significantly not only inter-individually but intra-individually in different time periods [12,17]. In addition, in the case of real-time PCR with intercalating dye, the amplification of FL-SMN and Δ7-SMN transcripts, as well as reference transcripts, was performed in separate reactions, which significantly increases the processing time and does not allow the simultaneous analysis of a large number of samples.

The most promising results were observed with semiquantitative and quantitative fluorescence RT-PCR, which enabled us to distinguish significant differences between SMA patients, SMA carriers, and healthy individuals in the mean percentage values of FL-SMN transcripts relative to the total sum of FL-SMN + Δ7 SMN transcripts values. These methods were shown to be convenient, fast, cost-effective, and reliable, demonstrating a high correlation in the obtained results. The mean percentage values of FL-SMN transcripts detected by this method were shown to increase in SMA cell cultures after treatment with therapeutic antisense oligonucleotides aimed at the correction of *SMN2* gene splicing. Concerning the in vivo application of the mean percentage of FL-SMN transcripts, we suggest that its usefulness is highly dependent on the drug type. We hypothesize that the assessment of FL-SMN percentage in blood cells of SMA patients treated by Nusinersen will not provide meaningful data because the drug is administered intrathecally, and whole-body distribution was not demonstrated [6]. However, it may be useful in onasemnogene-abeparvovec-treated patients because of the much wider biodistribution. According to a recent study of post mortem tissues of two SMA patients treated with onasemnogene abeparvovec, the AAV DNA and mRNA distribution were widespread among peripheral organs and in the CNS [18]. However, due to the fact that the virus rapidly reaches motor neurons in the spinal cord of SMA patients, it is doubtful that the intracellular *SMN* expression can be meaningfully measured in rapidly dividing blood cells. The most probable drug for which FL-SMN percentage measurement can be applied as a non-invasive biomarker is Risdiplam, which is a molecular modifier of *SMN2* gene splicing administered on a daily basis [19].

In conclusion, the results presented in this study show that the mean percentage of FL-SMN transcripts detected by semiquantitative and quantitative fluorescence RT-PCR can be used as a putative biomarker for the assessment of SMA therapy efficacy and for the development of new SMA drugs.

## Data Availability

The data presented in this study are available on request from the corresponding author. The data are not publicly available due to restrictions of the subjects’ agreement.

## Supplementary Materials

The following are available online at https://www.mdpi.com/article/10.3390/genes13101911/s1, Figure S1: Distribution of *SMN2* gene copy number between studied cohorts, Figure S2: Relative level of FL-SMN transcripts determined by semiquantitative and quantitative fluorescence RT-PCR-based methods.
