# Supplementary material for: Evaluation of Mean Percentage of Full-Length SMN Transcripts as a Molecular Biomarker of Spinal Muscular Atrophy
Source: Genes (Basel). 2022 Oct 20;13(10):1911. doi: 10.3390/genes13101911 (PMC9602009; doi:10.3390/genes13101911)
Supplement: Supplementary file 1 [file genes-13-01911-s001.zip › genes-1844530-supplementary.pdf]

Contents

Figure S1. Distribution of *SMN2* gene copy number between studied cohorts.............3

Figure S2. Relative level of FL-SMN transcripts determined by semiquantitative and quantitative fluorescence RT-PCR-based methods..4

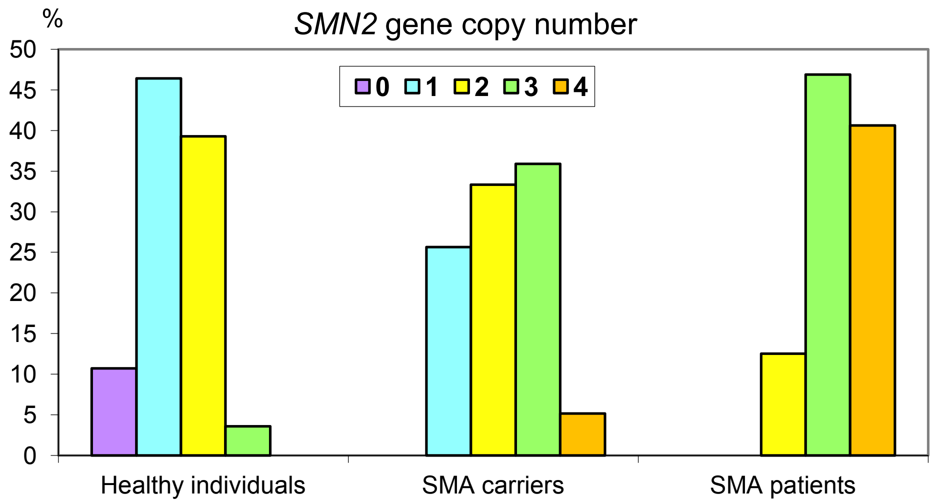

Figure S1. Distribution of *SMN2* gene copy number between studied cohorts.

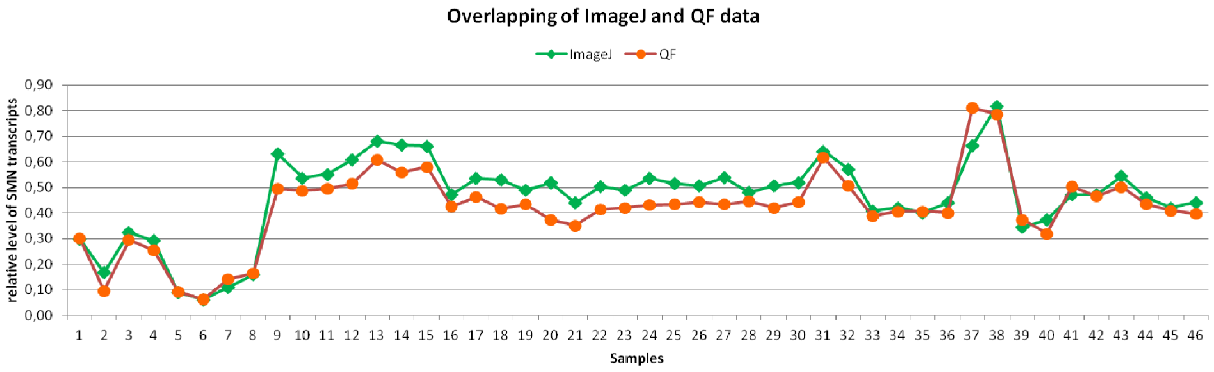

(a)

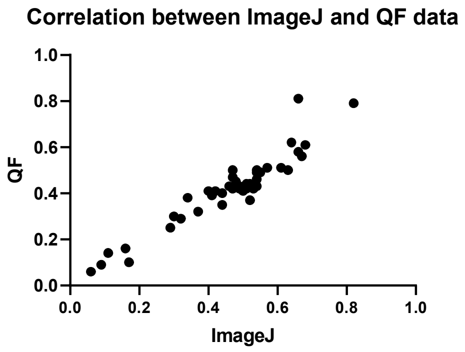

(b)

Figure S2. Relative level of FL-SMN transcripts determined by semiquantitative (ImageJ) and quantitative fluorescence (QF) RT-PCR-based methods. **(a)** – typical overlapping of data generated by both methods; **(b)** – correlation analysis between both methods.
